# GPU-FS-*k*NN: A Software Tool for Fast and Scalable *k*NN Computation Using GPUs

**DOI:** 10.1371/journal.pone.0044000

**Published:** 2012-08-28

**Authors:** Ahmed Shamsul Arefin, Carlos Riveros, Regina Berretta, Pablo Moscato

**Affiliations:** 1 Centre for Bioinformatics, Biomarker Discovery and Information-Based Medicine, The University of Newcastle, Callaghan, New South Wales, Australia; 2 Hunter Medical Research Institute, Information Based Medicine Program, John Hunter Hospital, New Lambton Heights, New South Wales, Australia; 3 Australian Research Council Centre of Excellence in Bioinformatics, Callaghan, New South Wales, Australia; UMR-S665, INSERM, Université Paris Diderot, Ints, France

## Abstract

**Background:**

The analysis of biological networks has become a major challenge due to the recent development of high-throughput techniques that are rapidly producing very large data sets. The exploding volumes of biological data are craving for extreme computational power and special computing facilities (i.e. super-computers). An inexpensive solution, such as General Purpose computation based on Graphics Processing Units (GPGPU), can be adapted to tackle this challenge, but the limitation of the device internal memory can pose a new problem of scalability. An efficient data and computational parallelism with partitioning is required to provide a fast and scalable solution to this problem.

**Results:**

We propose an efficient parallel formulation of the *k*-Nearest Neighbour (*k*NN) search problem, which is a popular method for classifying objects in several fields of research, such as pattern recognition, machine learning and bioinformatics. Being very simple and straightforward, the performance of the *k*NN search degrades dramatically for large data sets, since the task is computationally intensive. The proposed approach is not only fast but also scalable to large-scale instances. Based on our approach, we implemented a software tool GPU-FS-*k*NN (GPU-based Fast and Scalable *k*-Nearest Neighbour) for CUDA enabled GPUs. The basic approach is simple and adaptable to other available GPU architectures. We observed speed-ups of 50–60 times compared with CPU implementation on a well-known breast microarray study and its associated data sets.

**Conclusion:**

Our GPU-based Fast and Scalable *k*-Nearest Neighbour search technique (GPU-FS-*k*NN) provides a significant performance improvement for nearest neighbour computation in large-scale networks. Source code and the software tool is available under GNU Public License (GPL) at https://sourceforge.net/p/gpufsknn/.

## Introduction

The analysis of biological networks is an important task for gaining insights into the massive amount of data generated by high-throughput technologies (e.g., microarrays). Biological molecules such as proteins, genes, metabolites or microRNAs act as nodes in a biological network and the functional relationships between them are considered as edges. One essential task in the biological network analysis is to determine the nearest neighbours of some nodes of interest. When we are not given a network but a set of points in high-dimensional space, this simple problem actually turns into a computationally expensive optimization problem for finding the closest points of some query points in a metric space. Formally, for a set 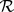 of 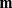 reference points and a set 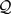 of 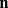 query points in a 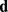-dimensional space, the *k*NN search problem identifies the *k*-nearest neighbours of each query point 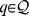 in the reference set 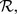 given a distance metric [1]. This method is commonly used in predictive analytics to classify a point based on the relationship to its neighbours. For example, if the majority of the neighbours of a query point belong to a certain class, then a verdict can be made that the query point *q* belongs to this class.

The *k*NN search problem arises in several fields of research, such as information retrieval, computer vision, databases, data compression, internet marketing, plagiarism detection, cluster analysis, etc. In 1973, D. E. Knuth called this problem, *the post-office problem*, referring it as the assignment of a residence to the nearest post office [2].

The basic *k*NN search technique is simple and straightforward and one can use an exhaustive search technique (also known as *brute force* approach) to find the nearest neighbours of a point. However, the actual computation of the distances and the nearest neighbours for large-scale instances requires a large number of computations. Several sequential approaches were proposed in [3], [4] to tackle this problem when an approximate solution is sufficient.

Interestingly, the basic brute force approach that gives the exact solution to this problem is highly parallelizable as the nearest neighbours of each query point can be computed and searched independently. This particular feature influenced us to create a GPU based data parallel solution for this problem. We have concentrated on a special case of the *k*NN-based optimization problem, in which the objective is to produce a *kNN graph* such that every node is connected to its *k*-nearest neighbours. The construction of the such graphs is an essential task in many fields of scientific research, such as data mining [5], [6], manifold learning [7], [8], robot motion planning [9], computer graphics [10] and bioinformatics [11]–[13]. We applied our proposed approach on microarray gene expression feature sets, considering the outcome can easily be integrated with existing graph-based clustering algorithms [14]–[16]. Furthermore, the basic idea can be adapted to scale the performance of other existing GPU-based *k*NN search methods [17], [18] and additionally, the implementation can be easily ported to other GPU architectures by incorporating simple modifications.

**Figure 1:**
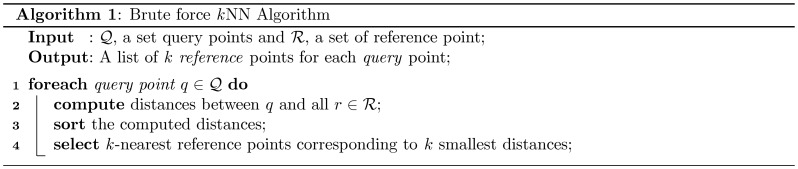
Pseudo-code for the Brute Force 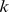
NN Algorithm. The run time complexity of the algorithm is 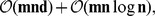 considering a total of 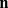 query points and 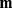 reference points in a 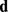-dimensional space.

### GPGPU Programming Model

The GPGPU is a powerful device that is devoted to parallel data processing rather than data caching and flow control as a general purpose CPU. Massive parallel processing capability of GPU makes it more attractive for algorithmic problem solving, where the processing of data (or a large block of data) can be handled in parallel. In general, the GPUs are organized in a streaming, data-parallel model in which the processors execute the same instructions on multiple data streams simultaneously. They are composed of a set of stream multi-processors (SM) with a certain number of stream processors (SP) each. Each SM contains a fast shared memory, which is shared by all of its processors. Additionally, a set of local registers is available for each SP (local memory). The typical sizes for shared memory are 16 K and 48 KB and local memory are 16 KB and 512 KB, depending on device compute capability [19]. The SMs communicate through the global/device memory which is much larger in terms of size but significantly slower than the other memory types (e.g., texture, constant, shared and local). The memory bandwidth and the peak floating-point capability of the GPU are much higher than the CPU. At the software level, there exist several programming interfaces (e.g., CUDA, OpenCL, DirectCompute or the most recent innovation like OpenACC) that enable programmers to develop applications on GPU. Among them, NVIDIA’s CUDA (Compute Unified Device Architecture) is one of the most widely used programming models that enable developing GPU-based applications using C/C++ programming language. Additionally, a number of third party CUDA wrappers are available for Python, Perl, Fortran, Java, Ruby, Lua, Haskell, MATLAB etc. CUDA exposes a higher level of abstraction to the programmers so that they can parallelize their tasks on GPU: a parallel task is instantiated as a collection of *threads*, organized in *blocks* (a 1, 2 or 3- dimensional collection of threads, where a limited amount of shared memory is available to all the threads in a block), arranged in a *grid* (a 1 or 2-dimensional collection of blocks). Maximum thread number in a block can be up to 1024 in the most recent architectures (e.g., “Fermi”) and maximum block number in a grid can be up to (2
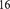
–1), in at most three dimensions. It should be noted here that *thread*, *block* and *grid* are CUDA specific terms. A CUDA program typically consists of a host component that runs on the CPU, or host, and a smaller, but computationally intensive device component called *kernel*, that runs in parallel on the GPU. The kernel cannot access the main memory of the host directly; input data for the kernel must be copied to the GPU’s on-board memory prior to its invocation, output data from the kernel must first be written to the GPU’s memory and then copied back to the host CPU memory.

**Table 1:** Summary of hardware, CPU and GPUs used for running our experiments.

Processor	CPU	GPU_A	GPU_B
Commercial Model	2× Xeon (Intel) E5506 Quad-Core	GeForce GTX 480	Tesla C2050
Number of Cores	2×8 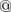 2.66 Ghz	15 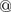 772 MHz	14 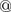 575 MHz
SIMD components	–	480 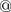 1.15 GHz	448 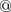 1.54 GHz
Memory Size	32 Gb (DDR3)	1.5 Gb (GDDR5)	4 Gb (GDDR5)
Interface to System	–	PCI-e ×16 Gen 2	PCI-e ×16 Gen

The Tesla C2050 used in this experiment is a commodity product in the marketplace with price ranging from USD $1,500
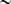
$ 2,000. It has 515 Gflops of double precision floating point performance (peak), 1.03 Tflops of single precision floating point performance (peak) and 144 GB/s memory bandwidth. On the other hand, GeForce GTX 480 is based on refreshed a “Fermi” architecture and much cheaper commodity product with price ranging from USD $500
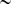
$600.

### The Brute Force *K*NN Search

The brute force approach computes similarity distances from each query point to all the reference points using a predefined metric (i.e. *Euclidean*, *Manhattan*, *Pearson’s*, *Spearman’s*, etc.). Then, the *k*-nearest neighbours are selected by sorting the distances. The complete method is described in Figure 1, Algorithm 1). Although the approach is very simple and straightforward, behind this apparent simplicity, there exists a high computational complexity. For instance, if we have a data set with 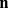 query points and 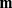 reference points in a 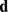-dimensional space, then, 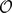
((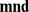)) time is required for the distance computation and 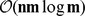 for sorting, therefore, a total 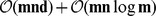 work is required for the complete computation. Now, to construct the *k*NN graph, we need to consider each data point both as a query point and a reference point (i.e., query 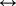 reference). Therefore, the 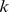
NN search needs to be performed on 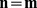 points and subsequently, the run time complexity becomes 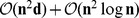. For a large number of points, the method can easily become prohibitive on general purpose computers. Fortunately, such distance computation and search can be performed independently for each query point. Therefore, one practical solution to improve the speed-ups is to parallelize the task. In the following section we explain the existing parallel brute force 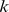
NN search methods.

### Existing Data-Parallel Approaches

A number of highly parallel approaches have been developed to reduce the computational overhead of the brute force 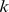
NN search problem. Garcia et al. [17] proposed the first GPU-based *k*NN searching algorithm that is at least 10 times faster than the sequential CPU implementation. The authors have demonstrated their algorithm with comb sort and insertion sort. Later, this implementation was studied by Nolan [20] and the author attempted to improve the performance of the algorithm using bitonic sort and a variant of bubble sort. The author also identified that the increase in the value of *k* dramatically decrease the performance of insertion sort based *k*NN implementation, while it remains comparatively stable with bitonic sort. Quansheng et al. [21] proposed a GPU-based implementation of brute-force *k*NN computation using the CUDA-based *radix sort* [22] that is at least 12 to 13 times faster than the sequential counterpart. They utilized a segmentation method for distance computation, which is similar to the distance computation method in [23], [24]. The implementation uses fixed size tiles (e.g., 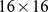) to construct the segments and the tile size depends on the available shared memory per CUDA blocks which is practically very limited (e.g., 16 KB or 48 KB) and hence, the method highly increases the number of data movements. Additionally, they proposed to classify the query points on CPU as it is difficult to optimize using GPUs. Liang et al. [18] proposed another CUDA-based parallel implementation of *k*NN algorithm, namely CU*k*NN, where they make use of streaming and coalesced data access for better performance. The implementation computes a set of distances by each CUDA block and outputs the local-*k* nearest neighbours and subsequently, it finds the global-*k* nearest neighbours when a set of blocks is finished. They achieved speed-ups of 21.81 times compared to sequential quick-sort based *k*NN and 46.71 times over an insertion sort based *k*NN. The implementation is suitable for small values of *k* up to 20, because of the limited amount of shared memory per CUDA block. Sun et al. [25] proposed a distributed approach for solving the *k*NN problem on large instances. They have proposed two layers of parallelization. In the first layer, which is an MPI-based layer, they distribute the data to several GPU enabled nodes. In the second layer, which is a CUDA layer, they compute the *k*-nearest neighbours for each query point. Finally, all the results are combined in the merging step. They have conducted tests on 96 nodes, where each node contains at least two GPUs and achieved speed-ups of over 80 times compared to the single GPU. However, the experiments are performed using a very expensive hardware i.e., on a supercomputer from NCSA (http://www.ncsa.illinois.edu) and limited values of 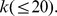 There exist many other parallel and distributed approaches to solve different special cases of the problem(see a detailed review in [26], [27]). However, many of the existing approaches often assume that the value of *k* is limited (e.g., Liang et al.[18], see the discussion above), the dimension of the data points is low (e.g., Connor et al.[26], the method is fairly scalable to large data sets but can work only with limited dimensions, 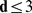) and sometimes the method itself is not scalable to very large instances (e.g., Garcia et al. [17], the method requires to compute and store a complete distance matrix of size 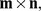 therefore, for large values of these variables (over 65,536), it becomes infeasible). In this work, we scaled and parallelized the simple brute force *k*NN algorithm (we termed our algorithm GPU-FS-*k*NN). It can typically handle instances with over 1 million points and fairly larger values of 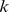 and dimensions (e.g., tested with 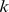 up to 64 and 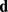 up to 295) on a single GPU. On multiple GPUs, if data partitioning is applied, then the method is capable of handling much larger instances and higher dimension sizes.

**Figure 2:**
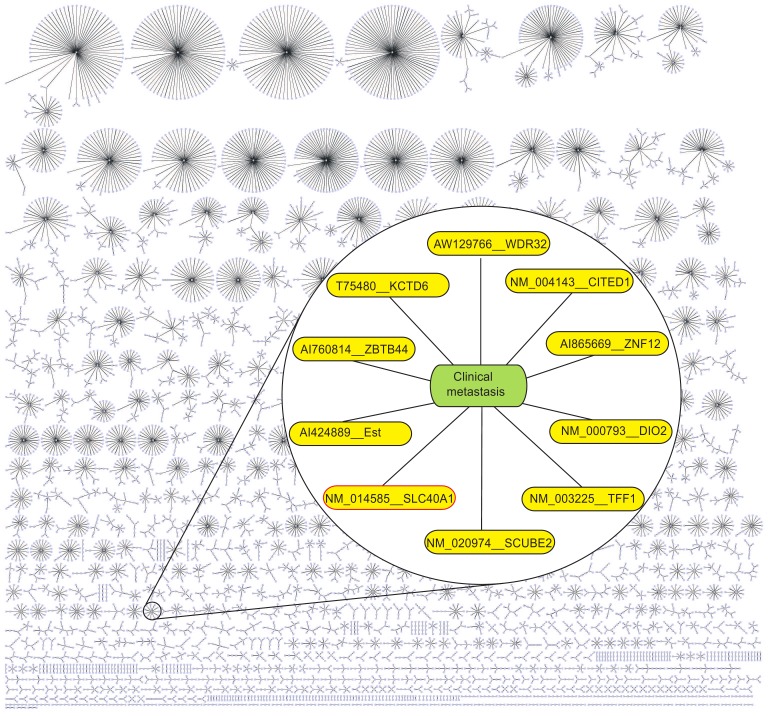
A *k*NN (for *k* = 1) graph from the breast cancer dataset with 24,158 probe sets in [28]. Zooming into the cluster containing only the *clinical metastasis*, brings out a list gene that are less highlighted in [28] but interesting enough to warrant further investigation. For instance, **Ferroportin** (solute carrier family 40 (iron-regulated transporter), member 1- SLC40A1) is one of less correlated gene in the original publication (see the Supplementary Table 3 (http://bioinformatics.nki.nl/data/nejm_table3.zip) of van de Vijver et al. [28]), but recent studies suggest that low level of Ferroportin increases breast cancer recurrence risk [39]–[42]. We utilized the freely available yEd software (http://www.yworks.com/) to visualize this graph.

**Table 2:** Running time comparisons for the *k*NN graph computation on GTX 480 (GPU_A) and Tesla C2050 (GPU_B).

Datasets	Size	1 CPU	16 CPUs	ChunkSize	1 GPU(A)	1 GPU(B)	4 GPUs(B)
original	24,158	60.22	10.58 (5.7x)	4,096	5.53 (10.3x)	5.18 (11.6x)	2.25 (26.8x)
				16,384	4.12 (14.6x)	3.25 (18.5x)	1.83 (32.9x)
				32,768	–	2.42 (27.4x)	1.22 (49.4x)
expanded_A	384,126	520.57	58.50 (8.9x)	4,096	42.57 (12.2x)	30.55 (17.1x)	15.28 (34.1x)
				16,384	30.25 (17.2x)	25.32 (20.6x)	12.15 (42.9x)
				32,768	–	18.65 (27.9x)	10.59 (49.2x)
expanded_B	1,533,876	1740.52	350.75 (4.8x)	4,096	88.48 (19.7x)	72.43 (24x)	40.57 (42.9x)
				16,384	79.53 (21.9x)	63.15 (27.6x)	36.84 (47.2x)
				32,768	–	54.37 (32x)	30.13 (57.8x)

The performance showed in terms of running times (in minutes) and speed-ups (x), on four different configurations, single threaded (1 CPU thread), multi-threaded (16 CPU threads), single GPU (GTX 480 (GPU_A) and Tesla C2050 (GPU_B), see Table 1) and multi-GPUs (4 Tesla C2050 GPUs). The time measurements are performed upon repeated executions of the method on each of these data sets and they include the times for loading and transferring data from the to and from the host and device memory. An increase in the chunk size (i.e., increased amount of computations on GPUs) performed better utilization of the parallel hardware and improved the overall the speed-ups. However, the execution times for the single and multi-core CPU implementations remained unchanged with chunk size variations due to the absence of computational chunking and higher chunk sizes could not be applied on GTX480 (GPU_A) due to its limited device memory (1.5 GB approximately). A total of 295 samples and a single value for 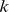 (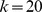) are used to perform these tests.

## Results

The computational tests are performed on following hardware setup: a total of four NVIDIA Tesla C2050 GPU cards are installed on a X8DTG-Q Supermicro server that has 2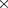 Intel Xeon E5620 2.4GHz processors, 32GB of 1066 MHz DDR3 RAM and 800GB of Local Hard Disk. To perform a fair comparison between speed-ups/cost ratio we also measured the performance on a GeForce GTX 480 (See Table 1 for the hardware details). The programs are written in C++ and CUDA (toolkit 4.0) and compiled using the g++ v4.4.4 and nvcc compilers on a Linux x86_64 version 2.6.9. The computational times are measured using CUDA timer utility [19].

### Preprocessing

We evaluate the performance of our proposed method on a renowned breast cancer gene-expression study data set provided by van de Vijver et al., [28] (see also van’t Veer et al. [29]). The original data set is available at http://bioinformatics.nki.nl/data.php, and has a total of 24,479 biological oligonucleotides and 1,281 control probes in 295 breast cancer patients. For this experiment we utilized the published log ratio of a total of 24,158 probe sets (mainly targeting genes) for all the 295 samples. The published clinical data gives the *clinical metastasis* (in terms of years to relapse for each patient); we consider this as a *phenotypical dummy probe* and keep it as a row (i.e., with the same status of a gene expression probe) in the input matrix. Our aim is to identify the nearest neighbors to this phenotype and derive a list of 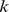 genes whose expression profiles closely match with the *clinical metastasis*. Note that this is possible because the measure used as metric (*Pearson’s correlation*) is insensitive to difference of scale between the two sequences (data for each probe) being compared.

**Table 3:** Running time comparisons of *k*NN computation for ANN-C++, BF-CUDA-*k*NN and GPU-FS-*k*NN on Tesla C2050.

Dim	Method	*n* = 10,000	*n* = 25,000	*n* = 50,000	*n* = 100,000	*n* = 1,533,876
8	ANN-C++	0.06	0.16	1.22	5.21	–
	BF-CUDA-*k*NN	0.01	0.09	0.25	–	–
	GPU-FS-*k*NN	0.01	0.15	0.51	1.15	20.52
16	ANN-C++	0.45	1.57	5.68	10.51	–
	BF-CUDA-*k*NN	0.01	0.11	0.29	–	–
	GPU-FS-*k*NN	0.01	0.21	0.65	1.48	21.25
20	ANN-C++	0.48	1.55	6.11	12.23	–
	BF-CUDA-*k*NN	0.01	0.15	0.35	–	–
	GPU-FS-*k*NN	0.01	0.25	0.68	1.55	21.43
32	ANN-C++	–	–	–	–	–
	BF-CUDA-*k*NN	0.01	0.25	0.81	–	–
	GPU-FS-*k*NN	0.01	0.40	1.14	1.87	23.56
64	ANN-C++	–	–	–	–	–
	BF-CUDA-*k*NN	0.02	0.65	2.22	–	–
	GPU-FS-*k*NN	0.03	0.78	2.15	3.65	25.52
96	ANN-C++	–	–	–	–	–
	BF-CUDA-*k*NN	0.03	0.82	2.53	–	–
	GPU-FS-*k*NN	0.05	1.12	3.75	4.53	26.76
295	ANN-C++	–	–	–	–	–
	BF-CUDA-*k*NN	–	–	–	–	–
	GPU-FS-*k*NN	0.12	1.25	6.52	7.58	28.95

Comparison of the computation times (in minutes) of three different methods, sequential ANN-C++ [4], BF-CUDA-*k*NN[17] and our proposed method GPU-FS-*k*NN (on multi-GPUs). All the GPU-based tests are performed on Tesla C2050 (GPU_B) and the elements are derived from the *expanded_B* data set. Here, 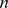 represents the data set size and the values of *k* are determined as, *k* =  i.e., 10, 11, 11, 12 and 15, respectively.

**Figure 3:**
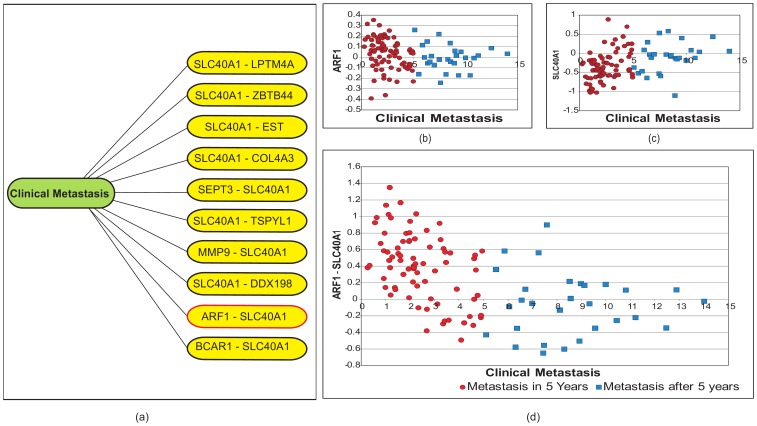
Application on the *expanded_A* dataset with 384,126 probe sets. Figure (a) shows of a *k*NN graph from the from *Expaneded_A* considering the *clinical metastasis* (year to relapse, a phenotypical “dummy” gene in the data set) as a sample query point. The multiple appearances of Ferroportin (SLC40A1) is noticeable in its neighboring metafeatures. The complete graph could not be visualized due to the limitation of existing tools [50]. Figure (b)–(c) show the correlation of ARF1 (ADP-ribosylation factor 1) and SLC40A1, (Ferroportin-1) with the the *clinical metastasis*, respectively. (d) The metafeature (ARF1-SLC40A1) shows better correlation with the *clinical metastasis* of each patient with respect to the feature (i.e., the ARF1, ADP-ribosylation factor 1, or SLC40A1, (Ferroportin-1) alone. This data indicates that, for those tumors that may relapse (and for which a different genetic signature may need to be found), the joint expression of ARF1 and Ferroportin may be associated to time to relapse.

To get a more diversified list and also to test the scalability of the proposed method, we extended our search space as follows: first, we filtered the probes sets using Fayyad and Irani’s algorithm [30]. This step is supervised and aims at finding differentially expressed probe sets in the samples labeled *metastasis* (relapsed in first five years) versus the ones that are labeled *non-metastasis* (relapsed after first five years). Next, we refined the selection of probe sets using the (alpha-beta)-*k*-Feature set method [31]–[35]. At this stage, we obtained a set of 876 filtered and refined expression profiles. Then, we produced two expanded data sets, one by applying the *difference
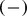
* operator between each possible pair of filtered probes and the other by applying four different operators: *difference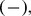 summation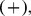 product
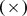
* and *division
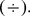
* These unique probe pairs are termed as *metafeatures* in Rocha de Paula et al. [36]. We call these two artificial data sets as *expanded_A* and *expanded_B*, containing 384,126 and 1,533,876 elements, respectively, where each of them has all the filtered probe sets and relevant metafeatures, for all 295 patients.

### Application

We applied the proposed method on each of these three data sets (original, expanded_A and expanded_B). A 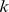
NN graph (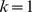) from the original data set is visualized in Figure 2 using the freely available yEd software (http://www.yworks.com/). Zooming into the cluster that contains the *clinical metastasis* (the *dummy probe*) brings out an interesting list of genes that are comparatively less highlighted in the original publication [28], but definitely warrant further investigation.

**Figure 4:**
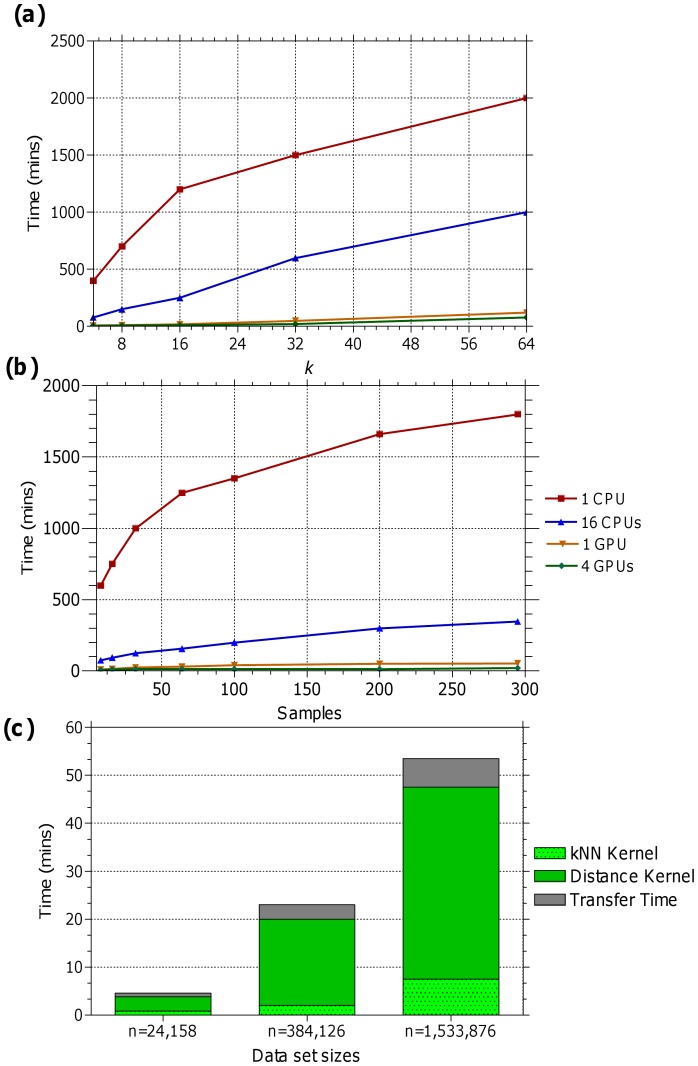
Performance of the GPU-FS-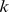
NN. Figure (a) and (b) show the computation times observed for different values of *k* (with a fixed sample size of 100) and sample sizes (with a fixed value of 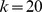), respectively, on the *expanded_A* data set. It is evident from these figures that even with an increase in the value of *k* or sample size, the execution time remains comparably stable on the GPU-based implementation. Figure (c) shows the computation times observed for the Distance kernel, kNN Kernel and data transfer from host to device on the three data sets, using a single GPU, 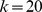 and a total of 295 samples. Although the distance computation takes most of the execution time here, further optimization to this kernel (e.g., vectorization) can improve the overall speed-ups.

For instance, **Ferroportin** (solute carrier family 40 (iron-regulated transporter), member 1- SLC40A1), which is one of the less highlighted genes in the original work (see the Supplementary Table 3 (http://bioinformatics.nki.nl/data/nejm_table3.zip), Vijver et al.[28]), showed interesting results in our experiments. When we applied the method (for 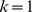) to the *expanded_A* data set and subsequently identified the cluster that contains the *clinical metastasis*, we not only found the multiple appearances of Ferroportin in several metafeatures (Figure 3(a)) but we also found certain metafeatures containing this particular gene to show better correlations with the *clinical metastasis*. For example, along with our previous investigation of (BCAR1 - SLC40A1) (see [15]), we found (ARF1 - SLC40A1) to show a better correlation than either the individual probe sets alone (e.g., genes ARF1 (ADP-ribosylation factor 1) or SLC40A1 (Ferroportin-1)). (Figure 3(b)–(d)). These results indicate that, for those tumors that may relapse (and for which a different genetic signature may need to be found), the joint expression of (ARF1) [37] or Breast Cancer Anti-estrogen Resistance 1 (BCAR1) [38] and Ferroportin (SLC40A1)[39]–[42] may be associated to the time to relapse. Application of the method to the *expanded_B* data set (not shown) also gave multiple appearances of Ferroportin in several metafeatures (with the *clinical metastasis*). In the same direction, other identified genes can be further investigated for their correlation with metastasis and breast cancer recurrence.

**Figure 5:**
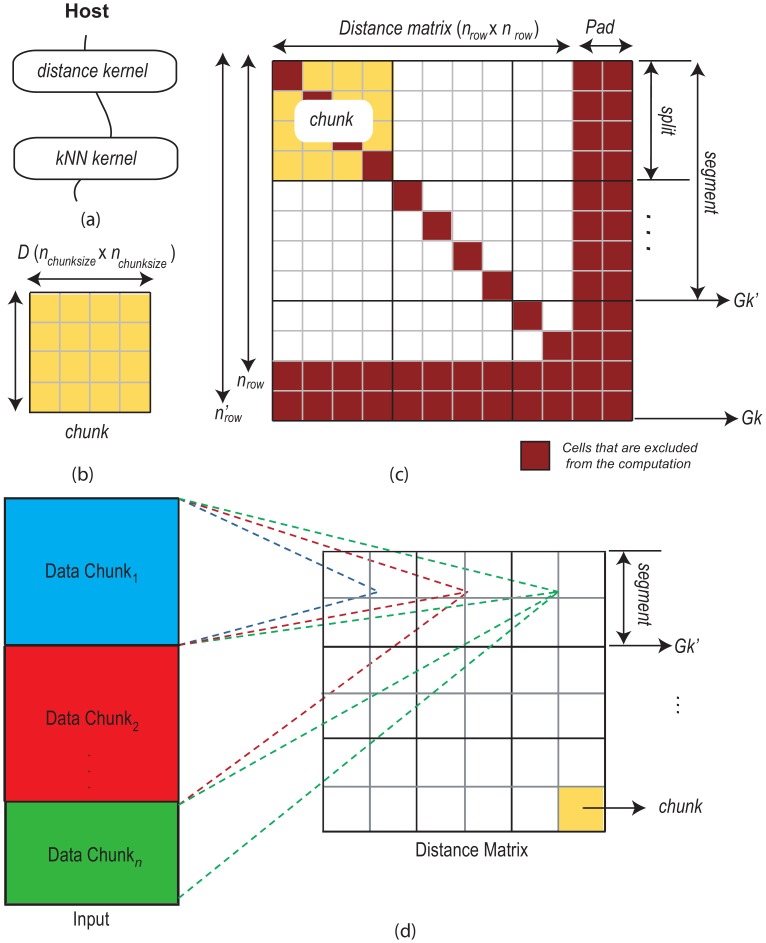
Basic principle of the proposed method. Figure (a) shows two kernels that execute sequentially, the first one creates the distance matrix and the second one identifies the nearest neighbours. Figure (b)–(c) shows the proposed method of computing *k*NN using chunk, split and segment of the original matrix. The original matrix is never kept in the device memory, each time a chunk is computed and respective 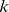
NNs are identified. Figure (d) shows an illustrative chunking of data for further scalability by splitting the data set horizontally into 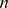 number of data chunks.

### Performance

The performances of the proposed method on two different GPUs, a GeForce GTX 480 (GPU_A) and a Tesla C2050 (GPU_B), over the CPU implementations (single core and multi-core) are demonstrated in Table 2. Our single threaded CPU implementation is based the brute force *k*NN algorithm (Figure 1, Algorithm 1) and the multi-threaded version is implemented by incorporating an OpenMP loop [43] inside the sequential implementation. We divide the total computation task in *chunks*, or piece of the total computation being submitted to the GPU as a single computational task (see Design and Implementation section for details). However, the proposed chunking method is not applied in these CPU implementations, as it may introduce further computational overhead. In each implementation, distances (similarities) computed using the *Pearson’s correlation* function.

**Figure 6:**
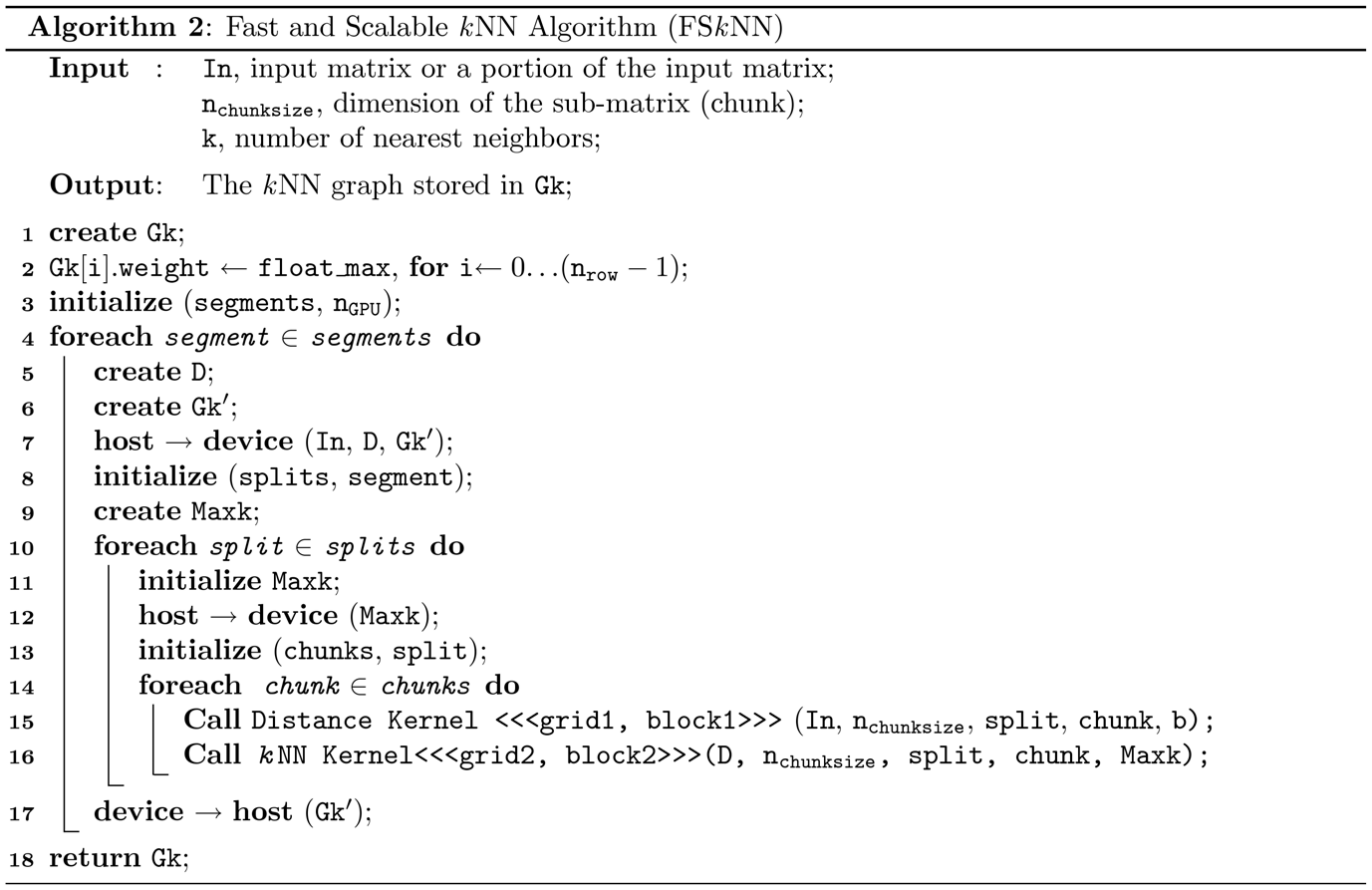
Pseudocode for the proposed scalable *k*NN search algorithm on GPU. The algorithm receives an input matrix (or a chunk of input matrix) In and produces a *k*NN graph ( Gk). It divides the complete distance matrix into small sub-matrices (“chunks”) and after computing all the chunks in a split, a partial 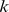
NN graph is derived which eventually becomes the final 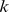
NN graph, when all the chunks in each split and all the splits in each segment are computed. The method has two level of parallelism, *chunk level* and *segment level*. The segment level parallelism is only applicable when the system has more than one GPU.

Performance-wise, during the single GPU execution, a maximum of 21.9× and 32× speed-ups are observed for the GPU_A and GPU_B, respectively. An increase in the chunk size (i.e., an increased amount of computations on the GPUs) increased the parallel hardware utilization and improved the overall speed-up. However, due to the limitation of the device memory, larger chunk sizes could not be applied on GPU_A. Additionally, multi-GPU tests are performed on the GPU_B only, since because the test system had only one GPU_A. The method is designed in a such way that an increase in the number of GPUs can further increase the execution speed-up. Therefore, we observed the maximum speed-up during the multi-GPU tests. Utilizing the largest chunk size (32,678), we obtained a maximum speed-up of 57.8× on the largest test data set (with over 1.5 M elements).

**Figure 7:**
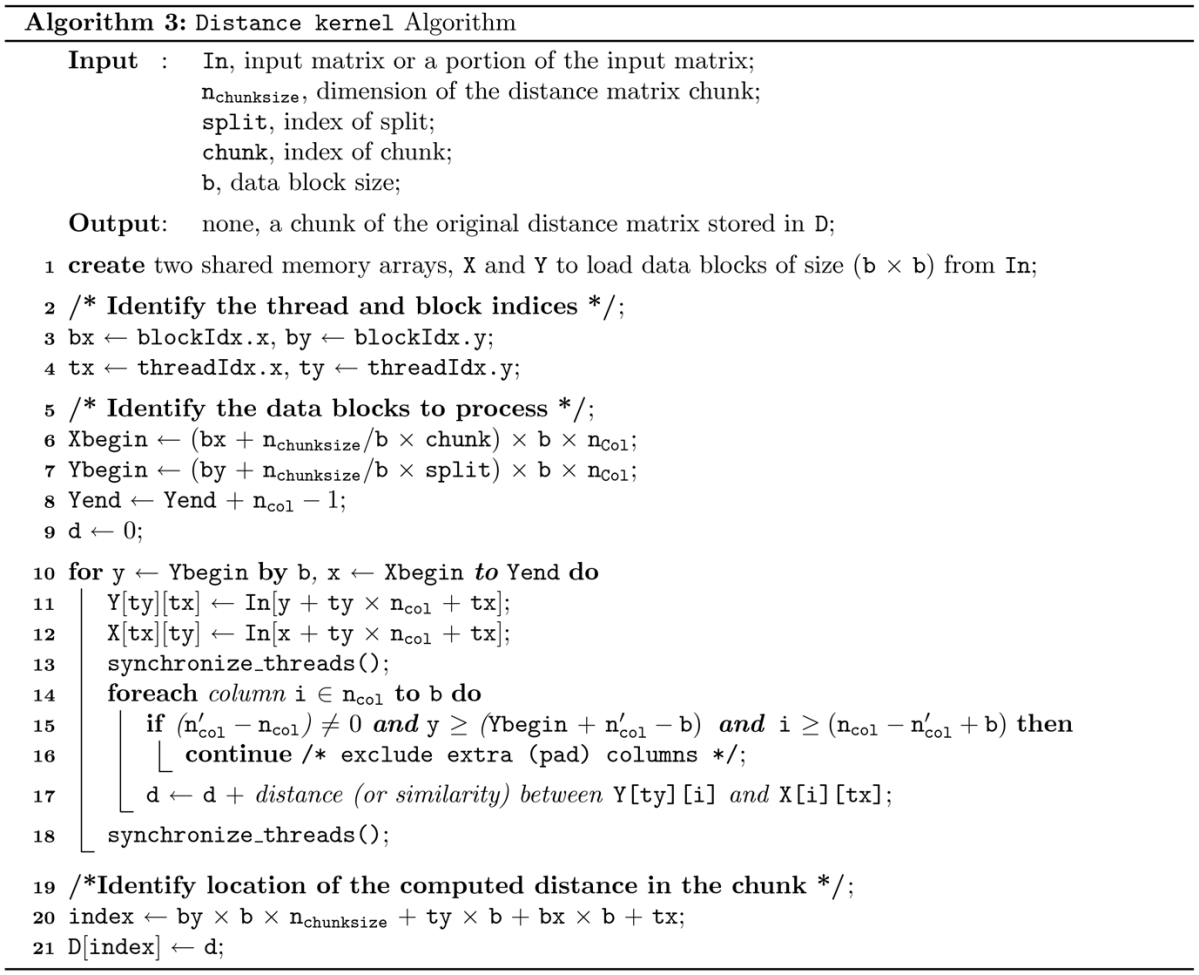
Pseudocode for the Distance Kernel. We present the Distance kernel as a template function which can be used for several types distance measures (such as *Euclidean* or *Manhattan* distance etc.) or similarity measures (such as *Pearson’s* correlation). Here, one thread is responsible for computing a single distance. The threads are organized as a set of two dimensional blocks and grids. Although the algorithm and the thread organization are adapted from Chang et al. [23], [24], we modified them to compute a chunk of the distance matrix instead of the complete distance matrix.

In Figure 4, we illustrate the performance of our proposed method for different values of 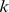 (with sample size of 100) and sample sizes (with fixed value of 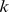 = 20) on the *expanded_A* data set. The Figures 4(a) and (b) depict that even with an increase in the value of 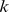 or sample size, the execution times remained comparably stable on the GPU-based implementation. In Figure 4 (c), we illustrate the execution times observed for the Distance kernel, kNN Kernel and data transfer from host to device. To measure this, we utilized the GPU_B, a fixed value for 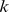 (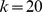) and all the 295 samples. This figure illustrates that the distance computation takes most of the execution time and further optimization to this kernel (e.g., vectorization) can improve the overall speed-ups.

**Figure 8:**
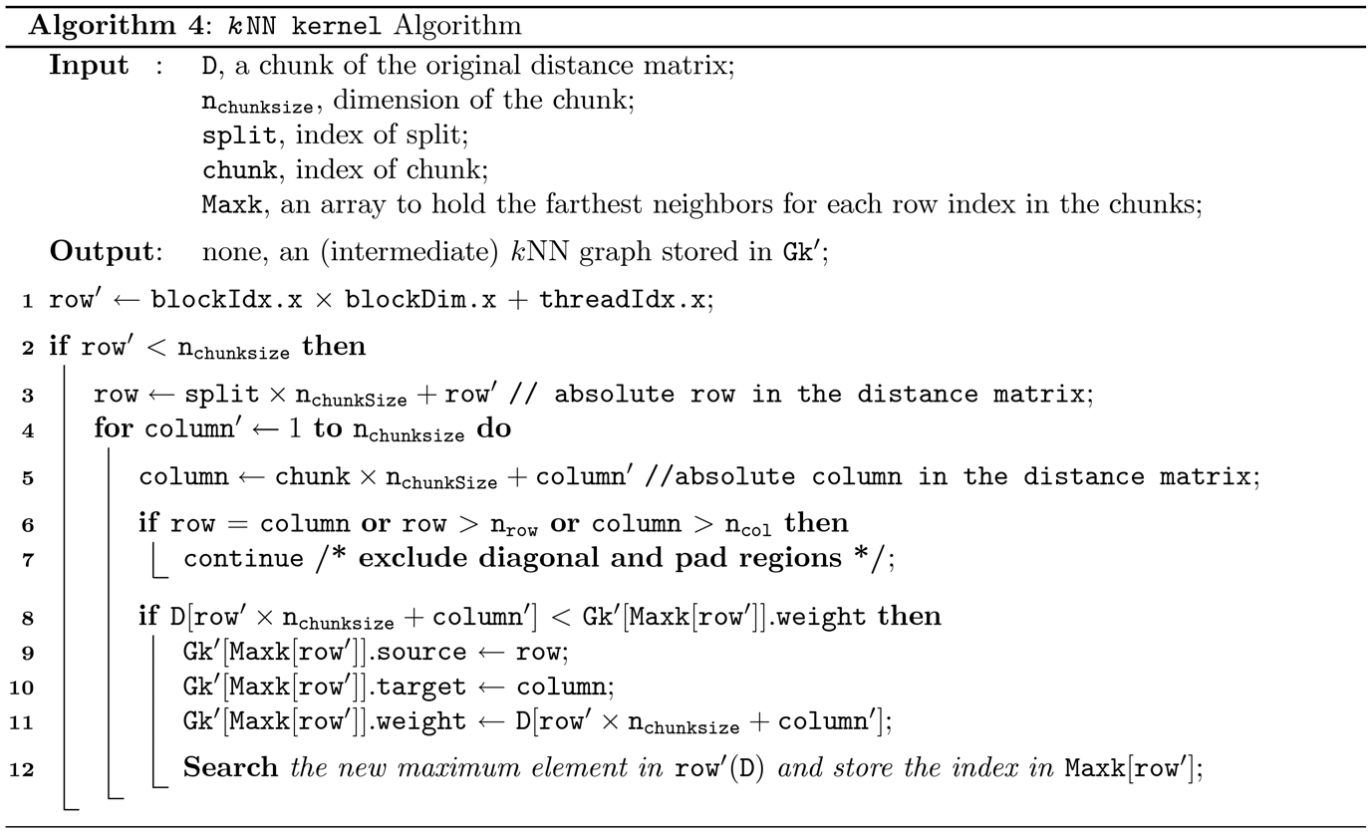
Pseudocode for the *k*NN Kernel. The kNN kernel algorithm utilizes one dimensional block and thread structure. Each thread works on a row of the chunk and identifies the *k*-nearest neighbors for each respective row index. Additionally, based on position of the chunk, it skips certain indices, e.g., the diagonal distance values (i.e., the distance from the point itself) and values that fall into the extra (padding) regions of the matrix.

In Table 3, we compare the performance of our proposed method with respect to two other known 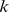
NN computation methods, a *k*−*d* tree based sequential approximate nearest neighbour (ANN-C++) computation [4] and a *bruteforce* algorithm based parallel *k*NN computation on GPU (BF-CUDA-*k*NN) [17]. In general, ANN-C++ is faster than our CPU implementation (not shown in table) but it could run only on a limited number of dimensions (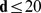) and the other GPU-based 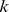
NN (BF-CUDA-*k*NN) executed slightly faster than ours but it could only work on a limited number of elements (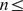 65,536, where 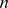 is the number of elements). On the other hand, the proposed approach could successfully perform the *k*NN computation on the largest test data set (1.5 M elements and 295 samples). These tests are performed using GPU_B and the elements derived from the *expanded_B* data set.

## Methods

The proposed method (GPU-FS-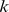
NN) operates on a simple partition and distribution of data and distance computation. In this work, we only discuss the details of the computational partitioning i.e., *chunking* of the distance matrix and subsequent identification method of 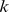
NNs from the chunks.

### Data Structures

The input data set is represented in the form of a matrix, where each row represents a point and the respective columns represent the dimensions of the point. The complete input matrix contains 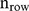 number of rows and 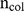 number of columns. Since, CUDA programming API does not support the transferring of multidimensional arrays from the host to device memory [19], we store the input matrix in a single dimensional array *In* of length (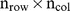), the distance matrix chunks in a single dimensional array D of length (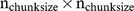), given a fixed chunk size, 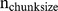 and the resultant *k*NN graph (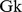) in an array of 3-tuples {source, target, weight} of length (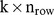). Additionally, we store the location of the farthest *k* nearest neighbours for each row index (chunk) in an array 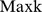 to facilitate the *k*NN search.

### Basic Principles

The proposed method has two different CUDA kernels that are executed one after another. The first kernel (Distance Kernel) calculates the sub-matrices (*chunks*) of the original distance matrix, where the second kernel (kNN Kernel) identifies the *k*NNs from from these chunks. When the original distance matrix completely fits into device in-memory, we consider the whole distance matrix as a single chunk (i.e., 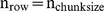), we simply execute the Distance kernel to produce the distance matrix and then, we invoke the kNN kernel to identify the *k*NNs list and subsequently we create the 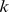
NN graph from the list (Figure 5(a)–(b)). In such case, only the value of 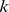 is required as an external parameter.

On the contrary, when the complete distance matrix is too large to fit into the device’s in-memory, we it break down into several chunks, where the size of the chunk (n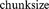) is provided as an external parameter, in addition to the value of 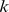. We consider all the chunks that share the same rows in the matrix are in a same *split* and we get a partial 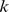
NN graph when all the chunks in a split are computed. Then, when all the splits in the distance matrix are computed, we get the complete 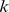
NN graph. However, at this point, we only achieve one level of parallelism i.e., in the *chunk level*. It possible to get another level by subdividing the splits into several *segments* and distributing them to separate GPUs. Therefore, we assign the number of GPUs as the number of segments and perform the segment’s computational tasks separately on each GPU. Same as previous, when all the splits in a segment are executed, we get a partial *k*NN graph and after the execution of all the segments, we get the complete the *k*NN graph. In Figure 5(c), we demonstrated the basic working procedure of the proposed computational chunking method.

### Fast and Scalable 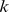
NN Search Algorithm

The proposed computational chunking is presented as an algorithm in Figure 6 (Algorithm 2). It starts with an input matrix (or a chunk of the input matrix, discussed later) In and produces a *k*NN graph (Gk). First, the weight attribute of each edge in the *k*NN graph is set to the maximum value of float (float_max) and the number of available GPUs (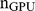) is assigned as the maximum number of segments (segments). The computational tasks for each of these segments are handled by separate GPUs. During the execution of each segment, the algorithm creates an array D (for holding a chunk of the matrix) and an additional *pointer* array 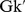 linked to 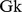 (for holding a partial *k*NN graph of the respective segment) and transfers them to the device memory. Now to compute the partial 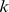
NN graph, the algorithm executes all the splits in each segment and subsequently all the chunks in each split. For each chunk the algorithm invokes the Distance kernel to compute a sub-matrix/chunk (D) and the kNN Kernel to compute an intermediate *k*NNs list. This list eventually becomes a partial 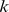
NN graph (stored in 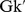) when all the chunks in a split and all the splits in a segment are executed completely. Then, the 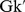 is transferred to host and mapped to the respective location in 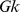.

We padded of the original distance matrix to fit all the chunks properly (Figure 5(c)). The padding of a matrix is a common practice in data-parallel task execution [44]. To do this, we extend the number of rows to 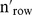 (Equation 1). Furthermore, for executing the Distance kernel on different chunk sizes, we extend the number of columns to 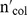 (Equation 2). It can be noted here that the chunk size should be a multiple of *data block size* b so that each CUDA block (in Distance kernel) can handle data blocks of size 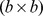 in parallel.
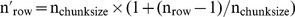
(1)

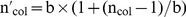
(2)


The basic approach (Figures 5 and 6) can be easily adapted to other GPU-based parallel architectures; however, the kernels need be transformed to appropriate functions to perform parallel computation of distance and 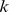
NN’s list.

### Computation of the Distance Kernel

The Distance kernel is presented as a template function in Figure 7 (Algorithm 3) which can be adapted for several types of distance measures, such as *Euclidean* or *Manhattan* distance or similarity measures that are based on *Pearson’s* or *Spearman’s* correlation. Here, each thread is responsible for computing a single distance in the matrix. The threads are organized in a 2-dimensional CUDA thread and block structure. Although the basic algorithm and the thread organization is adapted from Chang et al. [23], [24], we modified it to compute a chunk of the distance matrix instead of the complete distance matrix.

The working procedure of the algorithm is simple, during each iteration, every thread block loads (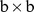) sized *data blocks* from the input matrix ( In) to two single dimensional shared memory arrays X and Y. Please note that the data in array X is loaded as the transpose of Y to reduce the number of bank conflicts in shared memory access[23]. After loading all the data blocks, each thread starts to calculate and accumulate its own partial distance in d. When the all the distances are finalized and threads are synchronized, each of them stores the value of d into the appropriate location in D.

It can be further noted that the algorithms in [23], [24] are designed to work only with the data sets where the number of rows and columns are multiples of 16 only (i.e., the *data block* size, b = 16). This limitation was imposed so that all threads in any half-warp (a *warp*  = 32 threads) can access the data in a sequence. We modified the original algorithm by introducing padded input matrix rows and columns (see Equation 1 and Equation 2) so that the algorithm can work with any number of rows and columns.

### Computation of the *K*NN Kernel

The kNN Kernel algorithm presented in Figure 8 (Algorithm 4) utilizes an 1-dimensional thread and block structure. Here, each thread works on a single row (chunk) and identifies the *k*-nearest neighbors for respective row index, where an array Maxk holds the location of the farthest *k*-neighbor. For each row index (chunk) the respective farthest *k*-neighbor is investigated and replaced if the distance to any element [*i*], 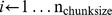 is found smaller. However, every index is not checked, rather based on the position of chunk in the original distance matrix the algorithm skips certain indices, for example, it excludes the diagonal indices (i.e., the distance from the point itself) for the chunks in diagonal positions and similarly it exclude the indices of the extra (pad) regions in the original distance matrix (Figure 5).

### Conclusions

The source code of the proposed GPU-based fast and scalable *k*-nearest neighbour search technique (GPU-FS-*k*NN) is available at https://sourceforge.net/p/gpufsknn/ under GNU Public License (GPL). Additionally, a part of the code is provided in the File S1 to demonstrate the CUDA kernels. The code can be compiled using NVIDIA CUDA compiler driver nvcc release 3.0 and up. It will require OpenMP support (“-fopenmp –lgomp”) to handle multiple GPUs and Boost library [45] to parse the input files. For convenience, we provided a makefile and a sample data set in the source code directory.

Outcome of our proposed tool can be used to generate approximate minimum spanning trees (AMST), minimum spanning forests (MSFs) [46] or clusters from large-scale biological data sets, such as microarrays[15]. The method is fairly scalable to large-scale data sets (tested with over 1.5 M elements). However, the main limitation of the current implementation is that it requires the complete data set to be in the device memory and when it is not possible to fit, the implementation becomes infeasible. Therefore, to improve the scalability, we plan to implement a *data chunking* along with the computational chunking of the distance matrix. A similar approach is presented in Li et at. [47] for computing large-scale distance matrices, where the authors have used a method similar to *Map-reduce* [48]. However, we propose to perform the chunking using external memory algorithms [49] so that the data can be handled even when it does not fit into host memory. One simplistic approach to achieve this could be splitting the input matrix horizontally into *n* number of data chunks (which could be in external memory for very large instances) and then sending a set of data chunks to the device for distance and 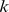
NN computations (see Figure 5(d)). Although the increased number of data transfers between the host and device can cause further slowdowns, a CUDA based *data streaming* (i.e., overlap of kernel execution and data transfer, see [19]) can be applied to reduce such transfer overhead.

## Supporting Information

File S1 Documented source code and description of the test data format.
(PDF)
