# Supplementary material for: GPU-FS-kNN: A Software Tool for Fast and Scalable kNN Computation Using GPUs
Source: PLoS One. 2012 Aug 28;7(8):e44000. doi: 10.1371/journal.pone.0044000 (PMC3429408; doi:10.1371/journal.pone.0044000)
Supplement: File S1 — Documented source code and description of the test data format. (PDF) [file pone.0044000.s001.pdf]

Supporting Information File S1

Documented Source Code

In this section we will discuss the source code of the GPU-based fast and scalable kNN (FSkNN) method. We performed our computational tests on the following hardware setup. Four NVIDIA Tesla C2050 GPU cards installed on a Xenon Nitro T5 Super-micro server that has Dual Xeon 5620 2.4GHz processors, 32GB of 1066 MHz DDR3 RAM and 800GB of Local Hard Disk. The programs were written in C++ and CUDA and compiled using the `g++` 4.4.4 and `nvcc` compiler on a Linux OS, Kernel version 2.6.9.

Source code compilation. The code can be compiled using NVIDIA CUDA compiler driver `nvcc` release 3.0 and up. It will require OpenMP support ("`-fopenmp -lgomp`") to handle multiple GPUs and Boost library to parse the input files.

Algorithm: GPU-FSkNN

Algorithm 1: Fast and Scalable kNN Algorithm (FSkNN)

```
Input  :  In, input matrix or a portion of the input matrix;
          n_chunksize, dimension of the sub-matrix (chunk);
          k, number of nearest neighbors;
Output:   The kNN graph stored in Gk;

create Gk;
Gk[i].weight ← float_max, for i← 0...(n_row − 1);
initialize (segments, n_GPU);
foreach segment ∈ segments do
    create D;
    create Gk′;
    host → device (In, D, Gk′);
    initialize (splits, segment);
    create Maxk;
    foreach split ∈ splits do
        initialize Maxk;
        host → device (Maxk);
        initialize (chunks, split);
        foreach  chunk ∈ chunks do
            Call Distance Kernel <<<grid1, block1>>> (In, n_chunksize,
            split, chunk, b);
            Call kNN Kernel<<<grid2, block2>>>(D, n_chunksize,
            Maxk);
    device → host (Gk′);
return Gk;
```

The algorithm starts with an input matrix `In` and produces a kNN graph (`Gk`). First, the `weight` attribute of each edge in the kNN graph is set to the maximum value of float (`float_max`) and the number of available GPUs (n_{GPU}) is assigned as the maximum number of segments (`segments`). The computational tasks for each of these segments are handled by separate GPUs. During the execution of each segment, the algorithm creates an array `D` (for holding a chunk of the matrix) and an additional pointer array `Gk′` linked to `Gk` (for holding the partial kNN graph of the respective segment) and transfers them to the device memory. Now to compute the partial kNN graph, the algorithm executes all the splits in each segment and subsequently all the chunks in each split. For each chunk the algorithm invokes the `Distance kernel` to compute a sub-matrix/chunk (`D`) and the `kNN Kernel` to compute an intermediate kNNs list. This list eventually becomes a partial kNN graph (stored in `Gk′`) when all the chunks in a split and all the splits in a segment are executed completely.Then, the `Gk′` is transferred to host and mapped to the respective location in `Gk`.

Implementation: fs$k$nn() function

```
/* fsknn function to represent the fsknn algorithm.
 * Input prameters:
 * @param filenameOut  : Output File name
 * @param Ina          : Input matrix (nRow x nCol)
 * @param nRow         : Total number of rows in the input matrix
 * @param nCol         : Total number of columns in the input matrix
 * @param chunkSize    : Total number of rows/columns in a chunk
 * @param k            : The integer value of k.
 * Output:
 * Graph(kNN) will be writen in (filenameOut).knn file in
 * the following format:
 *
 * number of vertices,  number of edges
 * source, target, weight
 * ....
 * source, target, weight
 */

int fsknn(char *filenameOut, float *Ina, int nRow, int nCol,
          int chunkSize, int k)
{
        /* define thread and blocks for Distance Kernel */
        dim3 blocksD (chunkSize/b,chunkSize/b);
        dim3 threadsD (b,b);

        /* define thread and blocks for kNN Kernel */
        int threadsK = 512; // maximum number of threads allowed
        int blocksK = chunkSize / threadsK+1;

        /* Declare local variables */
        float3 *Gka;
        int nRowExtended;
        int nColExtended;
        int nExtraCol;
        int nExtraRow;
        int splitSize;
        int segmentSize;

        /* Extend the columns */
        nColExtended = nCol;
        while (nColExtended % b !=0) {
            nColExtended++;
        }
        nExtraCol = nColExtended - nCol;


        /* Extend the rows */
```

```
        nRowExtended = nRow;
        while (nRowExtended % chunkSize !=0){
            nRowExtended++;
        }
        nExtraRow = nRowExtended - nRow;

        /* Identify total number of chunks in a split  */
        splitSize = nRowExtended / chunkSize;

        /* Allocate memory to store the kNN graph*/
        Gka = (float3) malloc (sizeof(float3) * nRowExtended * k);

        for (int i = 0; i < nRowExtended * k; i++)
                Gka[i].z = MaxValue; //initialize weights

        /* Setup multi-GPUs  */
        int nGpu = 0;
        cudaGetDeviceCount(&nGpu); //get the number of GPUs

        if(nGpu < 1){
                printf("no CUDA capable devices were detected!\n");
                return 0;
        }

        if(nGpu > splitSize) nGpu = splitSize; // for small matrices

        /* Identify total number of splits in a segment  */
        segmentSize = splitSize / nGpu;

        /* show some statistics on host and device */
        printf("number of host CPUs:\t%d\n", omp_get_num_procs());
        printf("number of CUDA devices:\t%d\n", nGpu);

        for(int i = 0; i < nGpu; i++)
        {
             cudaDeviceProp dprop;
             cudaGetDeviceProperties(&dprop, i);
             printf(" %d: %s\n", i, dprop.name);
        }

        printf("---------------------------\n");

        printf("#chunks in each split (splitSize): %d\n",splitSize);
        printf("#splits in each segment (segmentSize):%d\n",segmentSize);

        printf("---------------------------\n");
```

```
/* Implementation of the FSkNN algorithm   */

        /* Set the number of threads = the number of available GPUs */
        omp_set_num_threads(nGpu);

        /* outer loop, in parallel for each segment (GPU) */
        #pragma omp parallel
        {
             /* Host variables */
             float *Da;
             int *Maxka;
             float3 *Gka_sub; // a pointer  to the partial kNN graph
             unsigned int Gka_sub_bytes;

             /* Device variables    */
             float *dev_Ina,  *dev_Da;
             int *dev_maxArray;

             int tid = omp_get_thread_num();
             int numthread = omp_get_num_threads();
             int gpuid = -1;

             /* Set GPU by the thread id    */
             cudaSetDevice(tid);
             cudaGetDevice(&gpuid);

             /* Allocate memory on host */
             Da = (float*) malloc(sizeof(float)* chunkSize*chunkSize);
             Maxka = (int*)malloc(sizeof(int)*chunkSize);

             /* Allocate memory on device */
             cudaMalloc((void **) &dev_Ina,nRow*nCol*sizeof(float));
             cudaMalloc((void **) &dev_Da,
                        chunkSize*chunkSize*sizeof(float));
             cudaMalloc((void **) &dev_maxArray,
                        chunkSize*sizeof(int));

             /* Copy Ina[] and Da[] to  device */
             cudaMemcpy(dev_Ina, Ina, nRow*nCol*sizeof(float),
                        cudaMemcpyHostToDevice);
             cudaMemcpy(dev_Da, Da,chunkSize*chunkSize*sizeof(float),
                        cudaMemcpyHostToDevice);

             int splitBegin = tid * segmentSize;
             int splitEnd= splitBegin + segmentSize;
```

```
    /*For last split*/
    if (( splitSize - 1 )- splitEnd <= segmentSize )
        splitEnd = splitSize;

    Gka_sub = Gka + (tid*chunkSize*k)*(splitSize/numthread);

    /*  Calculate bytes used by the partial kNN graph */
    Gka_sub_bytes = (((tid+1)* chunkSize * k
                             * (splitEnd-splitBegin))
                             - (tid*chunkSize * k
                             * (splitEnd - splitBegin)))
                             * sizeof( float3);
    float3 * dev_Gka_sub = 0;
    cudaMalloc(( void **) &dev_Gka_sub,Gka_sub_bytes );
    cudaMemset (dev_Gka_sub,0,Gka_sub_bytes);
    cudaMemcpy (dev_Gka_sub,Gka_sub,Gka_sub_bytes,
                cudaMemcpyHostToDevice);

    /* Loop over all the splits in a segment*/
    for (int split = splitBegin; split < splitEnd ; split++){

       /* Copy Maxka[] to the device */
         for (int r=0;r<chunkSize;r++)
               *(Maxka + r) = (split - tid * segmentSize)
                               * k * chunkSize + r * k;
                cudaMemcpy (dev_maxArray, Maxka,
                            chunkSize * sizeof(int),
                            cudaMemcpyHostToDevice);

         /* Loop over all the chunks in a split*/
         for (int chunk = 0; chunk < splitSize; chunk ++){

               // Call Distance Kernel
               PearsonDistanceKernel <<<blocksD,threadsD>>>
               (dev_Ina,  dev_Da, chunkSize, split, chunk,
                nRow,nCol,nExtraCol, nColExtended);

               // Call kNN Kernel
               kNNKernel <<< blocksK, threadsK >>>
               (dev_Da,  dev_Gka_sub, dev_maxArray,
               chunkSize, nExtraRow, split, chunk, splitSize,
               nRow, tid,segmentSize, k);
         }
    }
   /*Copy back the partial kNN graph for a split*/
   cudaMemcpy(Gka_sub, dev_Gka_sub,
              Gka_sub_bytes,cudaMemcpyDeviceToHost);
```

```
/* Deallocate device memory */
            cudaFree(dev_Ina);
            cudaFree(dev_Da);
            cudaFree(dev_maxArray);
            cudaFree(dev_Gka_sub);
      }    // Close OpenMP loop


/*Write down the kNN graph into an external file (filename.knn)*/

          FILE * pFile;
          pFile = fopen (filenameOut,"w");

          if (pFile!=NULL)  {
                  fprintf(pFile,"%d %d\n",nRow, nRow * k);
                  for (int i = 0; i < nRow*k; i++)

                          // source, target and weight
                          fprintf(pFile,"%d %d %f\n",
                                 int(Gka[i].x),
                                 int(Gka[i].y),
                                 Gka[i].z);
                  fclose (pFile);
          }
         return 0;
}
```

Distance Kernel Algorithm

Algorithm 2: `Distance kernel` Algorithm

Input : `In`, input matrix or a portion of the input matrix;
$\mathtt{n}_{\mathtt{chunksize}}$, dimension of the distance matrix chunk;
`split`, index of split;
`chunk`, index of chunk;
`b`, data block size;

Output: none, a chunk of the original distance matrix stored in `D`;

```
create two shared memory arrays, X and Y to load data blocks of size
  (b × b) from In;
/* Identify the thread and block indices */;
bx ← blockIdx.x, by ← blockIdx.y;
tx ← threadIdx.x, ty ← threadIdx.y;
/* Identify the data blocks to process */;
Xbegin ← (bx + n_chunksize/b × chunk) × b × n_Col;
Ybegin ← (by + n_chunksize/b × split) × b × n_Col;
Yend ← Yend + n_col − 1;
d ← 0;
for y ← Ybegin by b, x ← Xbegin to Yend do
    Y[ty][tx] ← In[y + ty × n_col + tx];
    X[tx][ty] ← In[x + ty × n_col + tx];
    synchronize_threads();
    foreach column i ∈ n_col to b do
        if (n'_col − n_col) ≠ 0 and y ≥ (Ybegin + n'_col − b) and i
          ≥ (n_col − n'_col + b) then
            continue /* exclude extra (pad) columns */;
        d ← d + distance (or similarity) between data in Y[ty][i]
          and X[i][tx];
    synchronize_threads();
/*Identify location of the computed distance in the chunk */;
index ← by × b × n_chunksize + ty × b + bx × b + tx;
D[index] ← d;
```

Computation of the Distance Kernel. The `Distance kernel` is presented as a template function which be adapted for several types distance measures, such as *Euclidean* or *Manhattan* distance or similarity measures that are based on *Pearson's* or *Spearman's* correlation. Here, each thread is responsible for computing a single distance in the matrix. The threads are organized in a 2-dimensional CUDA thread and block structure. Although the basic algorithm and the thread organization have been adapted from Chang et al. [1, 2], we modified it to compute a chunk of the distance matrix instead of the

complete distance matrix. The working procedure of the algorithm is simple, during each iteration, every thread block loads (`b`×`b`) sized data blocks from the input matrix `In` to single dimensional shared memory arrays `X` and `Y`. Then after synchronization and each thread starts to calculate and accumulate own partial distances in `d` . When the distance values are finalized, each thread stores `d` to the appropriate location in `D`. It should be noted here that the algorithms in [1, 2] were designed to work only with the data sets where the number of rows and columns are multiples of 16 only (i.e., the *data block* size, `b`=16). This limitation was imposed so that all threads in any half-warp (a *warp* = 32 threads) can access the data in a sequence. We modified the original algorithm by introducing padded input matrix rows and columns so that the algorithm can work with any number of rows and columns.

Implementation: Distance Kernel ()

```
/* Distance kernel (Implements Pearsons Correlation).
 * This code has adapted from the paper "Compute pairwise Manhattan
 * distance and Pearson correlation coefficient of data points with
 * GPU" by Chang et al. (2009) but instead of computing the whole
 * distance matrix, we compute a chunk of size (chunkSize x chunkSize)
 * of the complete matrix.
 *
 * Input Parameters:
 * @param Ina          : Input matrix (nRow x nCol)
 * @param Da           : Distance matrix chunk (chunkSize x chunkSize)
 * @param chunkSize    : Total number of rows in a chunk
                         (must be multiple of b)
 * @param split        : split ID
 * @param chunk        : chunk ID
 * @param nExtraCol    : No. of extra columns added afterwards
 * @param nColExtended : Total number of columns in the
                         extended input matrix
 * Output:
 * none, Da[] left on the device memory
 */



// Computes Pearsons Correlation
__global__ void PearsonDistanceKernel(float *Ina, float *Da,
                int chunkSize, int split, int chunk, int nRow,
                int nCol, int nExtraCol, int nColExtended)
{

  __shared__ float Xs[b][b];
  __shared__ float Ys[b][b];

```

```
  int bx = blockIdx.x, by = blockIdx.y;
  int tx = threadIdx.x, ty = threadIdx.y;
  /* Adapted from Chang et al. (2009)  */
  int xBegin = (bx + (chunkSize/b) * chunk) * b * nCol;
  int yBegin = (by + (chunkSize/b) * split) * b * nCol;

  int yEnd = yBegin + nCol - 1;
  int x, y, i, index;

  float a1, a2, a3, a4, a5;
  float avgX, avgY, varX, varY, cov, rho;

  a1 = a2 = a3 = a4 = a5 = 0.0;
  for(y=yBegin,x=xBegin;y<=yEnd;y+=b,x+=b){

        /* load partial data into shared memory */
         Ys[ty][tx] = Ina[y + ty*nCol + tx];
         Xs[tx][ty] = Ina[x + ty*nCol + tx];
         __syncthreads();

         /* Calculate and accumulate partial similarity/distance */
         for(i = 0; i < b; i++ ){
            if (nExtraCol!=0
               && (y>=(yBegin +(nColExtended-b)))
               && (i>= nCol-(nColExtended-b)))
                  continue;

            a1 += Xs[i][tx];
            a2 += Ys[ty][i];
            a3 += Xs[i][tx] * Xs[i][tx];
            a4 += Ys[ty][i] * Ys[ty][i];
            a5 += Xs[i][tx] * Ys[ty][i];
        }
    __syncthreads();

  }
  avgX = a1/nCol;
  avgY = a2/nCol;
  varX = (a3-avgX*avgX*nCol)/(nCol-1);
  varY = (a4-avgY*avgY*nCol)/(nCol-1);
  cov  = (a5-avgX*avgY*nCol)/(nCol-1);
  rho  = cov/sqrtf(varX*varY);

  /* Identify the location (index) of the computed distance
  in the chunk of distance matrix */

  index = by*b*chunkSize + ty*chunkSize + bx*b + tx;
  Da[index] = rho;

}
```

*k*NN Kernel Algorithm

Algorithm 3: *k*NN kernel Algorithm

```
Input  :  D, a chunk of the original distance matrix;
          n_chunksize, dimension of the chunk;
          Maxk, an array to hold the farthest neighbors for each row
          index in the chunks;
Output :  none, an (intermediate) kNN graph stored in Gk';

row' ← blockIdx.x × blockDim.x + threadIdx.x;
if row' < n_chunksize then
    initialize row /* absolute index in the original distance matrix */;
    for column' ← 1 to n_chunksize do
        initialize column /* absolute index in the original distance matrix */;
        if row ← column or row > n_row or column > n_col then
            continue /* exclude diagonal and pad regions */;
        if D[row' × n_chunksize + column'] < Gk'[Maxk[row']].weight then
            Gk'[Maxk[row']].source ← row;
            Gk'[Maxk[row']].target ← column;
            Gk'[Maxk[row']].weight ← D[row' × n_chunksize + column'];
            Search the new maximum element in row'(D) and store the index in Maxk[row'];
```

Computation of the *k*NNs from a distance matrix chunk: The kNN Kernel algorithm utilizes an 1-dimensional thread and block structure. Here, each thread works on a single row (chunk) and identifies the k-nearest neighbors for respective row index, where an array Maxk holds the location of the farthest k-neighbor. For each row index (chunk) the respective farthest k-neighbor is investigated and replaced if the distance to any element$[i]$, $i \leftarrow 1 \ldots n_{chunksize}$ is found smaller. However, every index is not checked, rather based on the position of chunk in the original distance matrix the algorithm skips certain indices, for example, it excludes the diagonal indices (i.e., the distance from the point itself) for the chunks in diagonal positions and similarly it exclude the indices of the extra (pad) regions in the original distance matrix.

Implementation: kNN Kernel

```
/*
 *
 * Implements kNNKernel Algorithm
 *
 * Input Parameters:
 *
 * @param Da            : Distance matrix chunk (chunkSize x chunkSize)
 * @param Gka           : An array of 3-tuples {source,target,weight}
                          to store the kNN graph (Gka)
 * @param Maxka         : An array that contains the index of the
                          farthest nearest neighbours of
                          each node (in a chunk)
 * @param chunkSize     : Total number of rows in a chunk
 * @param nRow          : Total rows in the original distance matrix
 * @param nExtraRow     : Extra rows added to fit all the chunks
 * @param split         : split ID
 * @param chunk         : chunk ID
 * @param splitSize     : Total number of chunks in a split
 * @param chunkSize     : Total number of rows in a chunk
 * @param tid           : Openmp threadID (segment/gpu ID)
 * @param k             : Integer value of k
 *
 * Ouput:
 * none, kNN graph (Gka) will be left on the device.
 *
 *
 */

__global__ void kNNKernel (float* Da,  float3* Gka, int *Maxka,
                          int chunkSize, int nExtraRow, int split,
                          int chunk, int splitSize,  int nRow,
                          int tid, int segmentSize, int k)
{

/* Identify the row by thread id*/

int row = blockIdx.x*blockDim.x + threadIdx.x;

/* If the identified row belongs to the chunk*/
if (row < chunkSize)
   {

      /* Set the search range */
      int beginSearch = (split - tid * segmentSize) * k
                        * chunkSize + row * k ;
      int endSearch   = beginSearch + k;
      /*Search by Columns of the chunk*/
```

```
    for(int column=0;column < chunkSize ; column++){

        /* For Chunks in the diagonal of the original matrix */
        if(split == chunk){

           if(row != column){ //skip distance from the point itself

               /* Exclude the diagonal and extra regions
               of the matrix*/

               if (( nExtraRow !=0 )
                     && ((split >= splitSize - 1 )
                        || (chunk >= splitSize - 1 ))
                           && (row >= chunkSize - nExtraRow
                              || column >= chunkSize-nExtraRow ))
                                 continue;



               if(( *(Gka + *(Maxka + row ))).z >
                        *(Da + chunkSize * row + column )) {

                              ( *(Gka + *(Maxka + row))).z
                                 = *(Da + chunkSize * row + column );

                              ( *(Gka + *(Maxka + row))).x
                                 = split * chunkSize + row;

                              ( *(Gka + *(Maxka + row))).y
                                 = chunk * chunkSize + column;

                               /* Search the index of the max element
                               within range */
                               for (int index = beginSearch;
                                        index < endSearch;
                                        index++){

                                        if(( *(Gka+index)).z >
                                        (*(Gka + *(Maxka + row))).z )
                                           *(Maxka+row) = index;

                                        } // inner for loop

                     } //if

             }  // if row != column

        } // if split == chunk
        /* For chunks in non-diagonal positions of
           the original matrix */
```

```
        else
        {
             /* Exclude the extra regions of the matrix*/

            if ((nExtraRow != 0)
            && (column >= chunkSize - nExtraRow
              &&  chunk >= splitSize-1)
                 || (nExtraRow != 0)
                    && (row >= chunkSize - nExtraRow
                       && split>=splitSize-1))
                           continue;

                   if(( *(Gka +  *(Maxka + row))).z >
                        *( Da + chunkSize * row + column )){

                      ( *(Gka + *(Maxka + row))).z
                         = *(Da + chunkSize * row + column);

                      ( *(Gka + *(Maxka + row))).x
                         = split * chunkSize + row;

                      ( *(Gka + *(Maxka + row))).y
                         = chunk * chunkSize + column;


                     /* Search the index of the max element
                        within range */
                     for (int index = beginSearch;
                          index < endSearch;
                          index ++){

                          if((*(Gka+index)).z >
                             (*(Gka + *(Maxka + row))).z)
                                 *(Maxka + row) = index;
                         } // inner for loop

                    } // if

      }   // (else condition) i.e., if split != chunk

    }   // for loop ends

  }   // if row < chunkSize

}
```

Data Structures

The input data set is represented in the form of a matrix, where each row represents a point and the respective columns represent the dimensions of the point. The complete input matrix contains n_{row} number of rows and n_{col} number of columns. Since, CUDA programming API does not support transferring of multidimensional arrays from host to device memory, we store the input matrix in a single dimensional array In of length $(n_{row} \times n_{col})$, the distance matrix chunks in a single dimensional array D of length $(n_{chunksize} \times n_{chunksize})$, given a fixed chunk size, $n_{chunksize}$ and the resultant kNN graph (Gk) in an array of 3-tuples $\{source, target, weight\}$ of length $(k \times n_{row})$. Additionally, we store the location of the farthest k nearest neighbours for each row index and chunk in an array $Maxk$ to facilitate the kNN search.

```

/* An array of 3-tuples {source,target,weight} to store the kNN graph (Gka),
 this can  be replaced by a structure such as

        typedef struct
        {
                int source;
                int target;
                float weight;
        } Edge;
        Edge *Gka;

        */

        float3 *Gka;

        float3 *Gka_sub; // A pointer to Gka (for a segment)

        float *Ina; // Holds the input matrix
        float *Da;  // Holds chunk of the original distance matrix

        int k; // user defined value of k
        int chunkSize;  // user defined chunk size
        int *Maxka; // An array to hold the farthest value of k

        int nRow; //Total Number rows in the input matrix
        int nCol; //Total Number columns in the input matrix
```

Program Execution

We show a sample demonstration, input and output of our program in this section.

Input file format

Filename:Microarrays_CIBM_format.txt

```
<MicroarrayData>
10      6
F_1     1       4.5     2       7.3     8.21    5.71
F_2     1       3       8       6.66    7.19    1.06
F_3     3       3       9       5       6.67    7.73
F_4     4       4       1       4       3.64    2.92
F_5     8       7       1.5     4       4.91    6.15
F_6     8       6       3       2.4     7.36    4.76
F_7     3       8       3       3.2     5.31    5.49
F_8     2       1       4.02    5.8     3.08    7.15
F_9     4       1       3.7     2.2     1.31    1.48
F_10    -3      0       3       1       3.82    2.7
<SamplesNames>
        C1      C2      C3      C4      C5      C6
<SamplesClasses>
        1       1       1       0       0       0
<EndOfFile>
```

Running the Program

```
@mendel:./gpufsknn Microarrays_CIBM_format.txt

File Name (input)= TestData.bio.csv

Calculation starts at time : 17:15:49
Features (nRow) = 10  Samples(nCol) = 6
Data file loaded successfully...!
Type = Microarray
number of host CPUs:    16
number of CUDA devices: 1
   0: Tesla C2050
---------------------------
Total no of chunks in each split (splitSize):   1
Total no of splits in each segment (segmentSize):       1
CPU thread 0 (of 1) uses CUDA device 0
kNN computation finished at time : 17:15:55
Writing....output File Name = Microarrays_CIBM_format.txt.knn
```

Output file: Microarrays_CIBM_format.txt.knn

```
10 30
0 5 -0.145362
0 8 -0.782624
0 4 -0.117918
1 5 -0.480781
1 4 -0.866238
1 3 -0.415669
2 5 -0.486956
2 4 -0.763127
2 3 -0.833721
3 9 -0.520289
3 1 -0.415669
3 2 -0.833721
4 9 -0.673579
4 1 -0.866238
4 2 -0.763127
5 7 -0.620824
5 1 -0.480781
5 2 -0.486956
6 7 -0.317340
6 1 -0.292388
6 8 -0.834691
7 4 -0.367099
7 5 -0.620824
7 6 -0.317340
8 0 -0.782624
8 6 -0.834691
8 9 -0.426985
9 8 -0.426985
9 4 -0.673579
9 3 -0.520289
```

Conclusion

We developed a software tool based on our scalable approach for computing large-scale kNN graph using GPU and CUDA (FS-kNN-GPU). The source code is available under GNU Public License (GPL) at `https://sourceforge.net/p/gpufsknn/`

This is a hybrid approach where the host is responsible for loading, chunking and distributing the data and computation to the device, where the device uses two kernels to compute the distance and kNNs of each feature in the data set. The basic approach is simple and adaptable with other parallel frameworks. Outcome of our proposed tool can be used to generate approximate minimum spanning trees (AMST), minimum spanning forests (MSFs) [4] or clusters from large-scale biological data sets, such as microarrays (See [3]). We believe this work can provide compelling benefits for several domains of bioinformatics data mining.

References

[1] Chang D, Jones NA, Li D, Ouyang M, Ragade RK: Compute pairwise Euclidean distances of data points with GPUs. In *Proc. of the IASTED International Symposium on Computational Biology and Bioinformatics*, IASTED 2008:278–283.

[2] Chang D, Desoky AH, Ouyang M, C RE: Compute pairwise Euclidean distances of data points with GPUs. In *Proc. of the 10th ACIS International Conference on Software Engineering, Artificial Intelligence, Networking and Parallel/Distributed Computing*, ACIS 2009:501–506.

[3] Arefin AS, Inostroza-Ponta M, Mathieson L, Berretta R, Moscato P: Clustering Nodes in Large-Scale Biological Networks Using External Memory Algorithms. In *ICA3PP (2)*, *Volume 7017 of* Lecture Notes in Computer Science. Edited by Xiang Y, Cuzzocrea A, Hobbs M, Zhou W, Springer 2011:375–386.

[4] Arefin AS, Riveros C, Berretta R, Moscato P (2012) knn-borvka-gpu: A fast and scalable mst construction from knn graphs on gpu. In: Murgante B, Gervasi O, Misra S, Nedjah N, Rocha AMAC, et al., editors, ICCSA (1). Springer, volume 7333 of *Lecture Notes in Computer Science*, pp. 71-86.

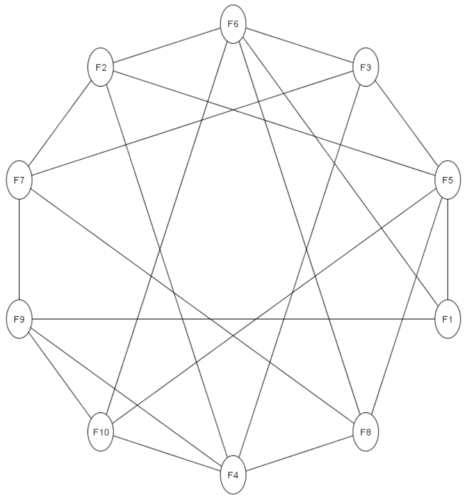

Figure 1: Results can be visualized using yEd (http://www.yworks.com) GML visualization tool. Visualization of the 3NN graph generated by the input file.

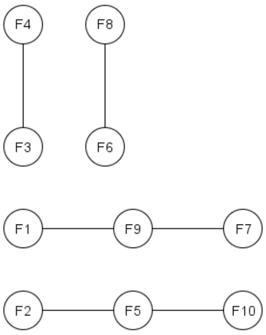

Figure 2: Visualization of the clustering created using the kNN graph and the MSTkNN+ algorithm in [3].
